# Incidence, predictors, and outcome of prosthesis-patient mismatch after transcatheter aortic valve replacement: A meta-analysis

**DOI:** 10.1097/MD.0000000000020717

**Published:** 2020-06-12

**Authors:** Shixin He, Zhenfei Fang

**Affiliations:** Department of Cardiology, the Second Xiangya Hospital of Central South University, China.

**Keywords:** incidence, outcome, predictors, prosthesis-patient mismatch, transcatheter aortic valve replacement

## Abstract

**Background:**

Prosthesis-patient mismatch (PPM) following transcatheter aortic valve replacement (TAVR) is common, but the incidence, predictors and outcome of PPM are still controversial.

**Methods:**

A total of 18 articles incorporating 72,016 patients were identified form PubMed and Embase online database.

**Results:**

The pooled incidences of overall, and severe PPM following TAVR were 32.0% and 10.0% separately. Comparing to surgical aortic valve replacement (SAVR), TAVR had lower incidence of overall (OR, 0.31, 95% CI, 0.20–0.50) and severe PPM (OR, 0.38, 95% CI, 0.28–0.52). PPM was associated with a larger body surface area (BSA), larger body mass index (BMI) and previous myocardial infarction in comparison with those patients without PPM. Although PPM was not rare after TAVR, no significant differences were observed both in short- and mid-term all-cause mortality (30 day: OR: 1.51, 95% CI, 0.79–2.87, 1 year: OR: 1.02, 95% CI, 0.96–1.08, and 2 years: OR: 0.99, 95% CI, 0.79–1.24) between patients with PPM and those without PPM.

**Conclusions:**

Despite the fact that the incidence of PPM was lower than that of SAVR, PPM was not seen to have an impact on short- and mid-term survival.

## Abstract 2

Supplemental Digital Content is available in the text

## 1 Introduction

Aortic stenosis is the most prevalent of all valvular heart diseases in developed countries, especially among old patients. In the Cardiovascular Health Study, which included 5201 men and women older than 65 years, a clear increase in prevalence of aortic stenosis was seen with age: 1.3% in patients aged 65 to 75 years, 2.4% in those aged 75 to 85 years, and 4% in patients older than 85 years. Patients with severe aortic stenosis have a terrible prognosis, with three-quarters dying within 3 years of symptom onset. The mean survival of patients with symptoms of aortic stenosis was remarkably increased in patients treated with aortic valve replacement vs those not undergoing this procedure.^[1]^ Initially, surgery was the only way for valve replacement and many patients who had been extremely ill from aortic valve stenosis and unresponsive to medical therapy were restored to good health by surgical aortic valve replacement (SAVR).^[2]^ However, there are still many problems after the surgery, prosthesis-patient mismatch (PPM) is 1 of them.

PPM is an indicator of the intrinsic relationship of the implanted valve to the cardiac output requirements of the patient.^[3]^ Prosthesis-patient mismatch occurs in the setting of a morphologically normal valve and is considered to be hemodynamically insignificant if the indexed EOA > 0.85 cm^2^/m^2^, moderate if between 0.65 and 0.85 cm^2^/m^2^, and severe if < 0.65 cm^2^/m^2^.^[4]^ Some studies stated that severe PPM is associated with increased short- and long-term mortality, worse post perioperative heart function, and less regression of left ventricular (LV) hypertrophy.^[5–10]^

Apart from the PPM, for patients with severe aortic stenosis who are not suitable candidates for surgery, transcatheter aortic valve replacement (TAVR) should be considered and recommended. TAVR could effectively reduce the rates of death and hospitalization, with a decrease in symptoms and an improvement in valve hemodynamics.^[11]^ With the prosperous development of techniques and prostheses, it is predictable that TAVR will be common among patients with severe aortic stenosis. Recently, more and more evidence also demonstrated that TAVR have comparable results in patients with intermediate surgical risk, compared with SAVR.^[12,13]^

Considering the potential damage of PPM, it is meaningful and important to study the PPM after TAVR. There are some studies that reported the relationship between PPM and TAVR, but the conclusions are controversial.^[14,15]^ Hence, we aimed to offer a meta-analysis to comprehensively and quantitatively investigate the incidence, predictors, and outcome of PPM after TAVR.

## 2 Methods

### 2.1 Literature search and study selection

**Figure 1:**
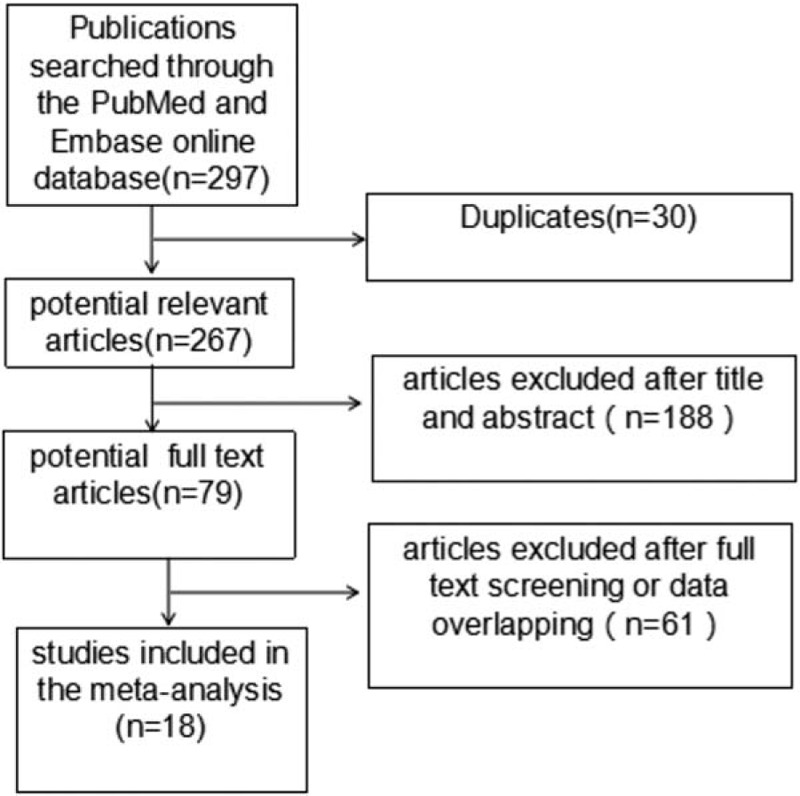
Flow diagram of citation research and selection.

Ethical approval and participants informed consent were not necessary because all data were extracted from previously published studies. The process of study selection was illustrated in Figure 1. The search strategy was described in supplementary material. The Articles were included if they

1. included the exact number or incidence of PPM;
2. defined the PPM as insignificant if the indexed EOA > 0.85 cm^2^/m^2^, moderate if between 0.65 and 0.85 cm^2^/m^2^, and severe if <0.65 cm^2^/m^2^;
3. indicated the predictive factors of PPM;
4. displayed the all-cause mortality of PPM;
5. were human adult studies and published in English.

The exclusion criteria were editorials, reviews, and case reports. There were 79 studies left after screening the titles and abstracts. Following full text screening and overlapped data removing, a total of 18 studies,^[14–31]^ incorporating 72,016 patients were eligible.

### 2.2 Data extraction

The 2 authors (Shixin He and Zhenfei Fang) independently extracted the data. The basic characteristics from eligible studies including author, year of publication, study location, patient baseline characteristics, the prevalence of PPM, and mortality analysis (Table 1). PPM in our meta-analysis was defined: moderate PPM (indexed EOA ≥ 0.65 cm^2^/m^2^ and ≤0.85 cm^2^/m^2^); severe PPM (index EOA < 0.65 cm^2^/m^2^).

**Table 1:**
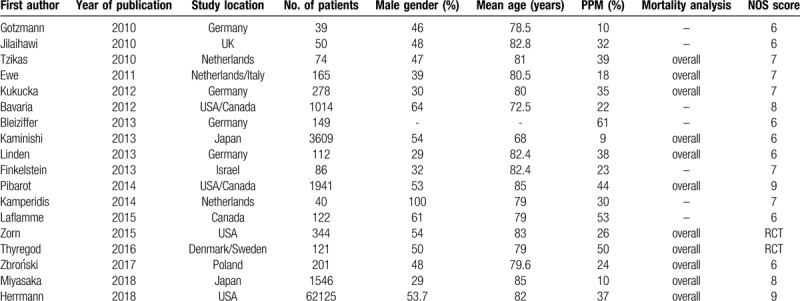
The study characteristics.

### 2.3 Quality assessment

The quality of eligible studies were assessed using the NOS scale (NOS score was listed in Table 1). Overall quality of these eligible studies was good.

### 2.4 Statistical analysis

Pooled incidences, odds ratios (OR), mean difference and risk difference were acquired using the Review Manager version 5.3. A random-effects model was used to obtain the pooled OR. Heterogeneity was assessed by calculating the *I*^2^ statistic. Publication bias was assessed by the Egger test in the meta-analysis. If the *P* value was less than .05, then publication bias existed.

## 3 Results

### 3.1 Incidence of PPM

The pooled incidences of overall, and severe PPM after TAVR were 32.0%, and 10.0% separately.

### 3.2 TAVR vs SAVR

**Figure 2:**
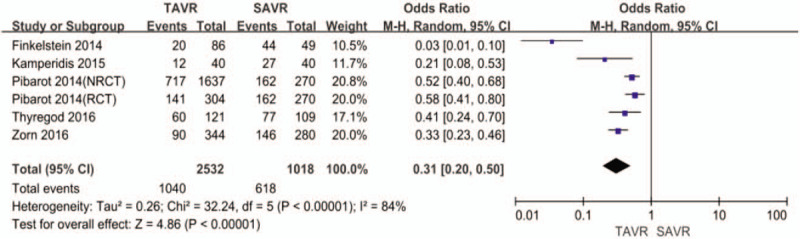
Odds ratio for overall prosthesis-patient mismatch comparing transcatheter aortic valve replacement with surgical aortic valve replacement.

**Figure 3:**
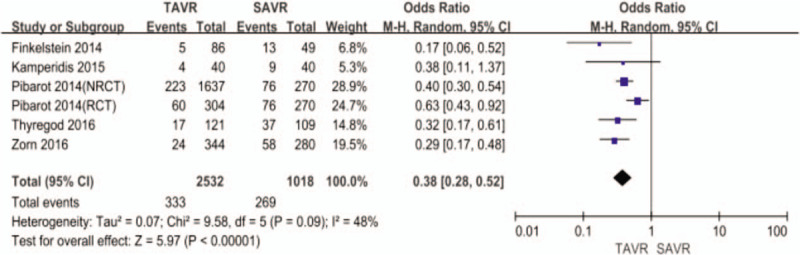
Odds ratio for severe prosthesis-patient mismatch comparing transcatheter aortic valve replacement with surgical aortic valve replacement.

TAVR had lower incidence of overall (41% vs 61%, OR: 0.31, 95% CI, 0.20–0.50, *I*^2^ = 84, *P* < .001, Fig. 2), and severe PPM (13% vs 26%, OR: 0.38, 95% CI, 0.28–0.52, *I*^2^ = 48, *P* < .001, Fig. 3) than SAVR. The Egger regression test suggested that significant publication bias was not observed in this meta-analysis (*P* = .062 for overall PPM, *P* = .308 for severe PPM) (Table 2). The Egger funnel plots were provided in supplementary Figures.

**Table 2:**
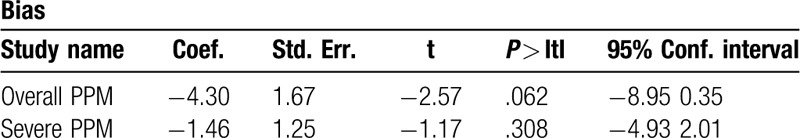
The Egger test of publication bias.

### 3.3 Predictive factors

**Figure 4:**
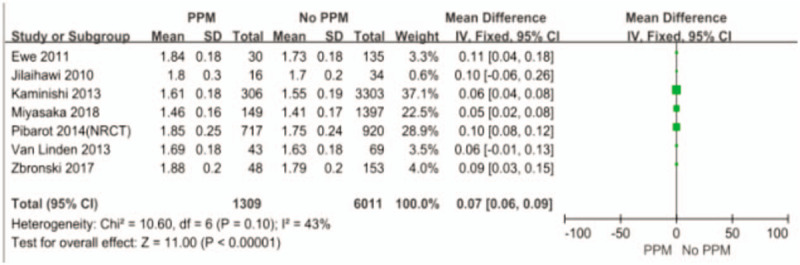
The difference of BSA between PPM and No PPM.

**Figure 5:**
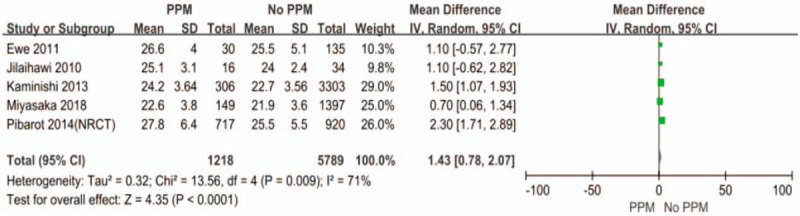
The difference of BMI between PPM and No PPM.

**Figure 6:**
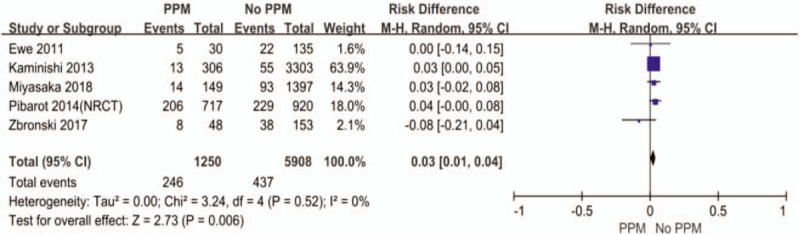
The difference of previous myocardial infarction between PPM and No PPM.

In order to investigate the predictors of PPM, we pooled some of the included studies using the univariate analysis method (Figs. 4–6). The differences of BSA, BMI, and previous myocardial infarction are statistically significant between PPM group and No PPM group. The PPM group was associated with larger body surface area (BSA), larger body mass index (BMI), and previous myocardial infarction.

### 3.4 Outcome of PPM

There was no significant difference between patients with PPM and those without PPM in both short-term and mid-term all-cause mortality (PPM vs No-PPM: 30 day: OR: 1.51, 95% CI, 0.79–2.87, 1 year: OR: 1.02, 95% CI, 0.96–1.08, and 2 years: OR: 0.99, 95% CI, 0.79–1.24) (Table 3).

**Table 3:**
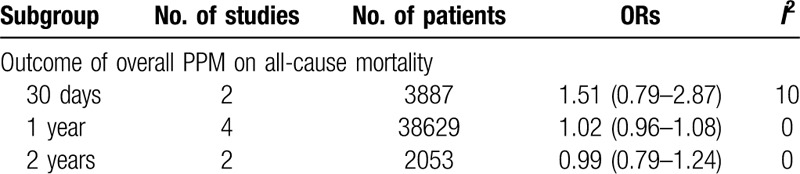
The outcome of PPM on all-cause mortality.

## 4 Discussion

The reported incidence of PPM after SAVR is diverse and ranging from 20% to 70%.^[26,32]^ The impact of PPM on patients prognosis is still controversial.^[3,33,34]^ There are some explanations that explain these discrepancies, for example:

1. different parameters used to define PPM and different methods used to estimate the EOA;
2. diverse types and sizes of prosthesis;
3. population heterogeneity.^[33]^

To overcome the above limitations of studies, meta-analysis is necessary. Promisingly, comparing to SAVR, TAVR was associated with lower risk in the prevalence of overall, moderate and severe PPM in our meta-analysis.

The pooled incidence of PPM following TAVR was 32%, while the prevalence of severe PPM was 10% in our meta-analysis. The definition of PPM in our eligible studies was based on measured EOA indexed to BSA. To evaluate the influence of PPM after TVAR more precisely, it is indispensable to standardize the measure of EOA (the data from in vivo, in vitro or by Doppler echocardiography). There is no doubt that invasive micromanometer catheter assessment of valves is the most accurate, but the application would be medically inappropriate after TAVR. In addition, considering the correlation between left ventricular output tract diameter (LVOTd) and EOA, the precise measurements of LVOTd is also vital for the reporting prevalence of PPM.

Now that PPM does exit in many patients after aortic valve replacement, we want to know the exact predictors of PPM, which may facilitate the clinical work. Larger BSA and BMI, previous myocardial infarction were the significant predictors in our meta-analysis. BSA and BMI are closely related to the choice of proper prosthesis and the calculation of PPM. Previous myocardial infarction is associated with poor vascular condition and increased risk of calcification of aortic valve, which may restrict the doctors from implanting a larger valve. Moreover, Dayan et al reported that female sex, older age, hypertension, diabetes, and renal failure were the main predictors for PPM.^[33]^ Therefore, to exactly determine the predictors of PPM, more precise and comprehensive patients information are needed.

Arguably, PPM after TAVR was not associated with increased short- and mid-term all-cause mortality in our meta-analysis, which was in accordance with the previous study.^[26]^ However, in some studies, severe PPM predicted higher mid-term mortality in a multivariable analysis.^[35,36]^ Several published studies, Takagi et al,^[37]^ Chen et al,^[38]^ and Head et al,^[3]^ reported a risk increase of 31%, 34%, and 42%, respectively, in mid and late all-cause mortality in patients with any degree of PPM. This paradox may be related to the absence of severe PPM subgroup in our analysis of outcome, the influence of individual preoperative characteristics and baseline comorbidities. Furthermore, our analysis included some newest large studies, which made it different from the others. Nonetheless, the influence of PPM on TAVR would be changeable with the development of new techniques and studies.

## 5 Limitations

There were several limitations that must be taken into account while interpreting the conclusions of the present meta-analysis. First, the included studies were small and mainly from America and Europe, so it would be more representative if patients from different continents are included. Second, studies focusing on severe PPM are still rare, therefore it is difficult to determine severe PPMs effect after TAVR. Third, although we tried our best to accomplish this meta-analysis, incomplete retrieval of identified research and reporting bias may be present.

## 6 Conclusion

TAVR in this study was associated with a significantly lower risk of overall, and severe PPM compared with SAVR. Although PPM after TAVR did not display a significant harmful effect on short- and mid-term all-cause mortality, it still seems reasonable to struggle to optimize TAVR hemodynamic performance and reduce the occurrence of PPM.

## Author contributions

**Conceptualization:** Zhenfei Fang.

**Data curation:** Shixin He, Zhenfei Fang.

**Methodology:** Shixin He, Zhenfei Fang.

**Supervision:** Zhenfei Fang.

**Writing – original draft:** Shixin He and Zhenfei Fang.

**Writing – review and editing:** Shixin He and Zhenfei Fang.

## Supplementary Material

Supplemental Digital Content

## Supplementary Material

Supplemental Digital Content
