# Supplementary material for: Incidence, predictors, and outcome of prosthesis-patient mismatch after transcatheter aortic valve replacement: A meta-analysis
Source: Medicine (Baltimore). 2020 Jun 12;99(24):e20717. doi: 10.1097/MD.0000000000020717 (PMC7302587; doi:10.1097/MD.0000000000020717)
Supplement: Supplemental Digital Content [file medi-99-e20717-s001.docx]

Search strategy

Pubmed:

Recent queries in pubmed

Search Query Items found Time

#4 Search ((((prosthesis patient mismatch[Title/Abstract]) OR PPM[Title/Abstract])) AND (((((percutaneous[Title/Abstract]) OR transcatheter[Title/Abstract]) OR transfemoral[Title/Abstract]) OR transapical[Title/Abstract]) OR transartery[Title/Abstract])) AND ((((aortic valve replacement[Title/Abstract]) OR aortic valve implantation[Title/Abstract]) OR AVR[Title/Abstract]) OR AVI[Title/Abstract]) Sort by: [pubsolr12] 183 8:33:31

#3 Search (((aortic valve replacement[Title/Abstract]) OR aortic valve implantation[Title/Abstract]) OR AVR[Title/Abstract]) OR AVI[Title/Abstract] Sort by: [pubsolr12] 20588 8:22:04

#2 Search ((((percutaneous[Title/Abstract]) OR transcatheter[Title/Abstract]) OR transfemoral[Title/Abstract]) OR transapical[Title/Abstract]) OR transartery[Title/Abstract] Sort by: [pubsolr12] 150483 8:20:38

#1 Search (prosthesis patient mismatch[Title/Abstract]) OR PPM[Title/Abstract] Sort by: [pubsolr12] 42770 8:18:50

Embase

Session Results

.......................................................

No. Query Results Results Date

#13. #2 AND #11 AND #12 114 6 Nov 2018

#12. #7 OR #8 OR #9 OR #10 32,955 6 Nov 2018

#11. #1 OR #3 OR #4 OR #5 OR #6 224,704 6 Nov 2018

#10. 'avi':ab,ti 841 6 Nov 2018

#9. 'avr':ab,ti 7,846 6 Nov 2018

#8. 'aortic valve implantation':ab,ti 9,071 6 Nov 2018

#7. 'aortic valve replacement':ab,ti 22,256 6 Nov 2018

#6. 'transartery':ab,ti 7 6 Nov 2018

#5. 'transapical':ab,ti 3,111 6 Nov 2018

#4. 'transfemoral':ab,ti 7,919 6 Nov 2018

#3. 'transcatheter':ab,ti 33,259 6 Nov 2018

#2. 'prosthesis patient mismatch':ab,ti 463 6 Nov 2018

#1. 'percutaneous':ab,ti 192,324 6 Nov 2018
