# Supplementary material for: Incidence, predictors, and outcome of prosthesis-patient mismatch after transcatheter aortic valve replacement: A meta-analysis
Source: Medicine (Baltimore). 2020 Jun 12;99(24):e20717. doi: 10.1097/MD.0000000000020717 (PMC7302587; doi:10.1097/MD.0000000000020717)
Supplement: Supplemental Digital Content [file medi-99-e20717-s002.docx]

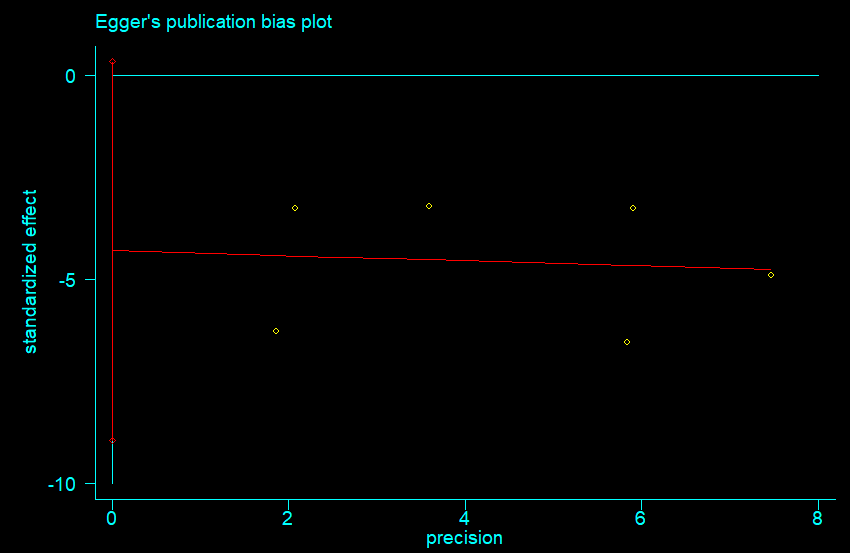


**Supplementary Figure 1.** Egger publication bias plot of overall PPM between TAVR and SAVR.


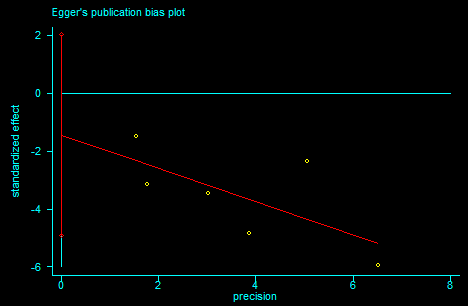


**Supplementary Figure 2.** Egger publication bias plot of severe PPM between TAVR and SAVR.
